# BBX11 promotes red light-mediated photomorphogenic development by modulating phyB-PIF4 signaling

**DOI:** 10.1007/s42994-021-00037-2

**Published:** 2021-04-26

**Authors:** Zhaoqing Song, Yueqin Heng, Yeting Bian, Yuntao Xiao, Jiujie Liu, Xianhai Zhao, Yan Jiang, Xing Wang Deng, Dongqing Xu

**Affiliations:** 1 State Key Laboratory of Crop Genetics and Germplasm Enhancement, National Center for Soybean Improvement, College of Agriculture, Nanjing Agricultural University, Nanjing, 210095 China; 2 Key Laboratory of Molecular Design for Plant Cell Factory of Guangdong Higher Education Institutes, Institute of Plant and Food Sciences, Department of Biology, School of Life Sciences, Southern University of Science and Technology, Shenzhen, 518055 China; 3 State Key Laboratory of Protein and Plant Gene Research, Peking-Tsinghua Center for Life Sciences, School of Advanced Agriculture Sciences and School of Life Sciences, Peking University, Beijing, 100871 China; 4 Biology Department, Brookhaven National Laboratory, Upton, NY 11973 USA

**Keywords:** phyB, BBX, PIF4, Photomorphogenesis, Light signaling

## Abstract

phytochrome B (phyB) acts as the red light photoreceptor and negatively regulates the growth-promoting factor PHYTOCHROME INTERACTING 4 (PIF4) through a direct physical interaction, which in turn changes the expression of a large number of genes. phyB-PIF4 module regulates a variety of biological and developmental processes in plants. In this study, we demonstrate that B-BOX PROTEIN 11 (BBX11) physically interacts with both phyB and PIF4. BBX11 negatively regulates PIF4 accumulation as well as its biochemical activity, consequently leading to the repression of PIF4-controlled genes’ expression and promotion of photomorphogenesis in the prolonged red light. This study reveals a regulatory mechanism that mediates red light signal transduction and sheds a light on phyB-PIF4 module in promoting red light-dependent photomorphognenesis.

**Supplementary Information:**

The online version contains supplementary material available at 10.1007/s42994-021-00037-2.

## Introduction

Sunlight consists of a wide range of wavelength spectrum which are perceived by at least five classes of photoreceptors in plants (Galvao and Fankhauser 2015; Paik and Huq 2019). Photo-activated photoreceptors promptly transduce light information to downstream signaling pathway, eventually promoting various light-mediated physiological and developmental processes (Paik and Huq 2019; Jing and Lin 2020; Wei et al. 2020; Yadukrishnan et al. 2020; Yadukrishnan and Datta 2020; Yang et al. 2020). Among these, skotomorphogenesis (etiolation) and photomorphogenesis (de-etiolation) are tightly dependent on the absence or presence of light signals at the seedling stage. In darkness, germinated seeds undergo skotomorphogenetic development exhibiting elongated hypocotyls, curved apical hooks and closed cotyledons with etiolated plastids. While in the light, seedlings display expanded cotyledons with well-developed chloroplasts and shortened hypocotyls designated as photomorphogenesis (Jiao et al. 2007; Xu 2020). These two distinct developmental processes and their transition are precisely under the control of light signal transduction pathway involving a variety of photoreceptors, kinases, E3 ubiquitin ligase complexes and transcription factors (Paik and Huq 2019; Xu 2020; Han et al. 2020; Jing and Lin 2020; Yadav et al. 2020; Yu and Liu 2020).

phytochrome A and B (phyA and phyB) are red and far-red light photoreceptors responsible for sensing a specific wavelength region from approximately 600 to 750 nm (Sharrock and Quail 1989; Li et al. 2011). Of these, phyB is the predominant red light photoreceptor that initiates a plethora of red light-mediated cellular and physiological events in plants (Li et al. 2011; Paik and Huq 2019). phyB resides in the cytosol and maintains at pr form which is biologically inactive in the dark. Upon red light irradiation, pr state of phyB is converted into biologically active pfr form, subsequently shifting into the nucleus from the cytosol. pfr state of phyB interacts with a subset of PHYTOCHROME INTERACTING FACTORS (PIFs: PIF1, PIF3, PIF4 and PIF5) and triggers their rapid phosphorylation, ubiquitination and degradation within minutes (Li et al. 2011; Pham et al. 2018; Paik and Huq. 2019). Although phyB promptly promotes the degradation of PIF4 at the early stage upon red light exposure, the abundance of PIF4 maintains at a high level under the prolonged red light conditions, suggesting that PIF4 protein re-accumulates in the prolonged red light which is necessary and essential for normal seedling growth (Park et al. 2018; Yan et al. 2020). Consistently, *pif4* mutant seedlings show shorter hypocotyls, whereas transgenic seedlings over-expressing *PIF4* exhibit longer hypocotyls than wide-type (WT) in the constant red light (Huq and Quail. 2002). As a b-HLH type transcription factor, PIF4 directly binds to the promoter regions of multiple regulators of auxin biosynthesis and signaling to promote cell elongation and hypocotyl growth in plants (Franklin et al. 2011; Sun et al. 2012, 2013). Thus PIF4 represents a key node in phyB signaling through which plants integrate diverse internal and external cues to modulate seedling development.

B-box proteins (BBXs), which are characterized by the presence of one or two conserved B-box domains at the N-terminal region, play critical roles in light signaling, photoperiodic flowering, pigment accumulation and distinct hormonal signaling pathways (Gangappa and Botto. 2014; Vaishak et al. 2019; Song et al. 2020a; Xu. 2020). There are 32 BBXs members in *Arabidopsis* which are classified into five subfamilies according to their respective domain structures and features (Khanna et al. 2009). Increasing studies have revealed that a group of BBXs regulate photomorphogenic development. BBX4 and BBX21-BBX23 are positive regulators of light signaling, whereas BBX19, BBX24, BBX25 and BBX28-BBX32 inhibit photomorphogenesis (Datta et al. 2006, 2007, 2008; Holtan et al. 2011; Fan et al. 2012; Gangappa et al. 2013; Wang et al. 2015; Xu et al. 2016, 2018; Zhang et al. 2017a, b; Job et al. 2018; Lin et al. 2018; Heng et al. 2019a,b; Yadav et al. 2019; Bursch et al. 2020; Song et al. 2020b; Wu et al. 2020). BBX4 specifically promotes phyB-mediated signaling (Datta et al. 2006; Heng et al. 2019a). Red light-activated phyB interacts with and stabilizes BBX4 that forms inactive heterodimers with PIF3 to repress its biochemical action and PIF3-controlled gene expression, thereby promoting photomorphogenesis in the red light (Heng et al. 2019a). BBX19 facilitates the CONSTITUTIVELY PHOTOMORPHOGENIC 1 (COP1)-mediated degradation of EARLY FLOWERING 3 (ELF3) which is a core member of the evening complex, consequently leading to the increase of *PIF4* and *PIF5* as well as hypocotyl growth (Wang et al. 2015). Unlike BBX4 and BBX19, the other BBXs form a transcriptional regulatory network with ELOGATED HYPOCOTYL 5 (HY5) by affecting its transcription and/or biochemical activity to negatively or positively regulate photomorphogenic development (Datta et al. 2007, 2008; Holtan et al. 2011; Fan et al. 2012; Gangappa et al. 2013; Xu et al. 2016, 2018; Zhang et al. 2017b; Lin et al. 2018; Heng et al. 2019b; Yadav et al. 2019; Bursch et al. 2020; Song et al. 2020b). As such, different BBXs are considered to exert distinct modes of action in the control of photomorphogenesis (Vaishak et al. 2019; Xu 2020; Song et al. 2020a).

Recently, we have revealed that BBX11, BBX21 and HY5 form a feedback loop that promotes photomorphogenesis. Loss of BBX11 function mutants are specifically hyposensitive to the monochromatic red light, but not to the blue and far-red light (Zhao et al. 2020), suggesting that BBX11 is a key component of phyB signaling. Here, we demonstrate that BBX11 physically interacts with both phyB and PIF4. BBX11 not only promotes the degradation of PIF4 but also inhibits its biochemical activity in the red light. Consequently, BBX11 modulates the phyB-PIF4-mediated signaling to promote photomorphogenic development in plants.

## Results

### BBX11 interacts with red light photoreceptor phyB

**Fig. 1:**
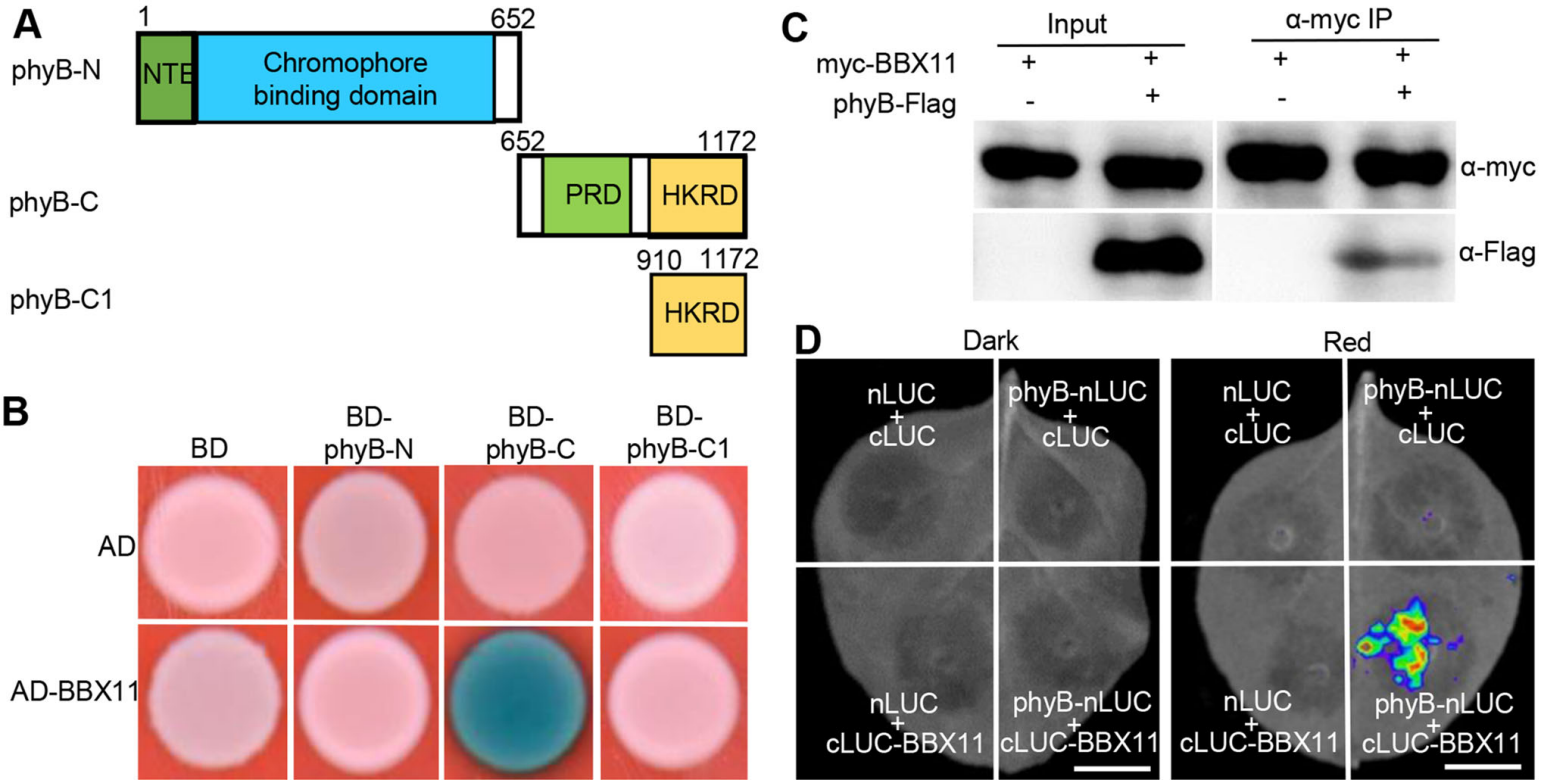
phyB interacts with BBX11 both in yeast and living plant cells. **A** Schematic diagram of various constructs used in the yeast two-hybrid assays. Numbers indicate the amino acid positions in phyB. **B** Yeast two-hybrid interactions between the various truncated phyB and BBX11 proteins. **C** Co-immunoprecipitation analysis showing that myc-BBX11 interacts with phyB-Flag. Total protein was extracted from wild tobacco leaves transiently expressing 35S:myc-BBX11 alone or together with 35S:phyB-Flag. The immunoprecipitates were detected using anti-myc and anti-Flag antibodies. **D** LCI assays showing the interaction of phyB with BBX11. Full-length phyB and BBX11 were fused to the split N- or C- terminal (nLUC or cLUC) fragments of LUC. The phyB-nLUC and cLUC-BBX11 were transiently co-expressed in *Nicotiana benthamiana* leaves and incubated in darkness for 1 day. Then the *Nicotiana benthamiana* plants were shifted in darkness or red light (94 μmol/m^2^/s) respectively for an additional 2 d. nLUC and cLUC were used as negative controls. Bar = 1 cm

Our previous studies suggest that BBX11 is a positive regulator of phyB signaling (Zhao et al. 2020). To explore the mechanism and molecular context of BBX11 action in this process, we first performed yeast-two hybrid experiments to test whether BBX11 directly interacts with red light photoreceptor phyB. As BINDING DOMAIN (BD)-fused phyB exhibited self-activation activity in yeast cells (Supplemental Fig. 1), we thus constructed BD-phyB-N (1–652 aa) containing a chromophore binding domain, BD-phyB-C containing a PRD and a HKRD domain (652–1172 aa) for this assay (Fig. 1A). ACTIVATING DOMAIN (AD)-fused BBX11 could interact with BD-phyB-C (652–1172 aa) but not BD-phyB-N (1–651 aa) (Fig. 1B). The phyB C-terminus contains one PRD and one HKRD domain. To examine the exact domain in phyB responsible for the association with BBX11, BD-phyB-C1 containing a HKRD (910–1172 aa) and BD-phyB-C2 containing a PRD (652–910 aa) were generated (Fig. 1A; Supplemental Fig. 1A). BD-phyB-C2 (652–910 aa) showed self-activation activity (Supplemental Fig. 1B), and BD-phyB-C1 (910–1172 aa) did not interact with AD-BBX11 in yeast cells (Fig. 1B). These results suggest that the C-terminal region of phyB is required for the interaction with BBX11. To verify the phyB-BBX11 interaction, we employed a co-immunoprecipitation (Co-IP) assay and transiently co-expressed 35S:myc-BBX11 alone or together with 35S:phyB-Flag in the *Nicotiana benthamian* leaves. myc-BBX11 co-immunoprecipitated phyB-Flag as detected by immunoblot analysis (Fig. 1C). Next, we performed firefly luciferase complementation imaging (LCI) assays and fused spilt N-terminal LUC (nLUC) and C-terminal LUC (cLUC) with phyB (phyB-nLUC) and BBX11 (cLUC-BBX11) respectively. The phyB-nLUC and cLUC-BBX11 were transiently co-expressed in *Nicotiana benthamiana* leaves. The *Nicotiana benthamiana* plants were kept in darkness for 1 d, and then transferred to darkness or red light for additional 2 d. We detected the strong LUC influence signals in *Nicotiana benthamiana* leaves with red light irradiation, but not in those leaves kept in darkness (Fig. 1D). The LUC signals were not detectable when transiently co-expressed negative controls nLUC and cLUC, phyB-nLUC and cLUC, or nLUC and cLUC-BBX11 in the *Nicotiana benthamian* leaves (Fig. 1D). Together, these results suggest that red light photoreceptor phyB associates with BBX11 in yeast and living plant cells, and the association of phyB with BBX11 might be dependent on the presence of red light.

### BBX11 physically interacts with PIF4

**Fig. 2:**
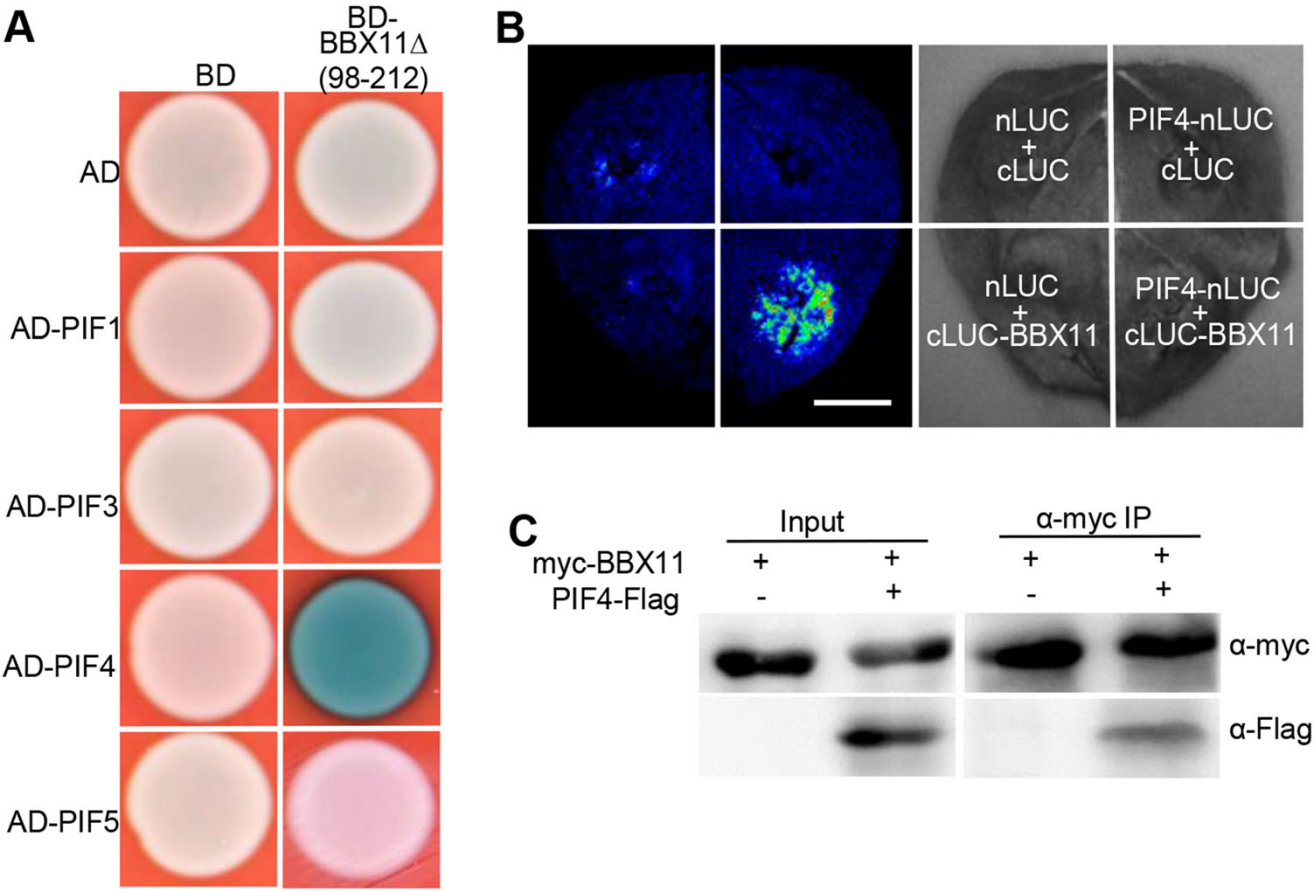
BBX11 physically interacts with PIF4. **A** Yeast two-hybrid assays showing that BBX11∆ (98–212) lacking transactivation domain interacts with PIF4, but not PIF1, PIF3 and PIF5. **B** LCI assays showing the interaction of PIF4 with BBX11. Full-length PIF4 and BBX11 were fused to the split N- or C- terminal (nLUC or cLUC) fragments of LUC. nLUC and cLUC were used as negative controls. Bar = 1 cm. **C** Co-immunoprecipitation analysis showing that myc-BBX11 interacts with PIF4-Flag. Total protein was extracted from wild tobacco leaves transiently expressing 35S:myc-BBX11 alone or together with 35S:PIF4-Flag. The immunoprecipitates were detected using anti-myc and anti-Flag antibodies

Photo-activated phyB directly interacts with downstream signaling components PIFs in mediating a variety of light-dependent development in plants (Leivar and Monte 2014), we thus examined whether BBX11 associates with PIFs. BBX11, which is a transcription factor, exhibited self-transactivation activity in yeast cells and its middle portion is likely responsible for this action (Supplemental Fig. 2), which is consistent with previous studies that the middle portion of BBXs possesses transcriptional activation activity (Zhang et al. 2014; Heng et al. 2019a; Bursch et al. 2020). We thus generated a BD-BBX11Δ(98–212 aa) construct lacing transactivation activity (Supplemental Fig. 2). Yeast-two hybrid assays showed that BBX11Δ(98–212 aa) specifically interacted with PIF4, but not with PIF1, PIF3 and PIF5 (Fig. 2A). LCI experiments showed that co-expression of PIF4-nLUC and cLUC-BBX11 in *Nicotiana benthamiana* leaves clearly produced LUC signals (Fig. 2B).When we transiently co-expressed negative controls nLUC and cLUC, PIF4-nLUC and cLUC, or nLUC and cLUC-BBX11 in *Nicotiana benthamiana* leaves, the LUC signals were not observed in the same detection system (Fig. 2B). Co-IP assays were employed to further verify these results. 35S:myc-BBX11 alone or together with 35S:PIF4-Flag were transiently expressed in the *Nicotiana benthamian* leaves. As shown in Fig. 2C, myc-BBX11 could pull down PIF4-Flag. Taken together, these results suggest that BBX11 physically interacts with PIF4 *in planta*.

### BBX11 promotes the degradation of PIF4 in the prolonged red light

To assess whether phyB regulates the abundance of BBX11, we introduced the *phyb-9* mutation into *YFP-BBX11* #8 by genetic crossing. *YFP-BBX11 #8* and *YFP-BBX11 #8 phyb-9* grown in the constant red light accumulated similar YFP-BBX11 abundance (Supplemental Fig. 3A). In addition, comparable YFP-BBX11 protein levels were detected in the dark-grown *YFP-BBX11 #8* and *YFP-BBX11 #8 phyb-9* upon transferred to red light irradiation for various time periods (0, 15, 30 and 60 min) (Supplemental Fig. 3B). These results indicate that phyB may not affect the accumulation of BBX11 in the red light.

**Fig. 3:**
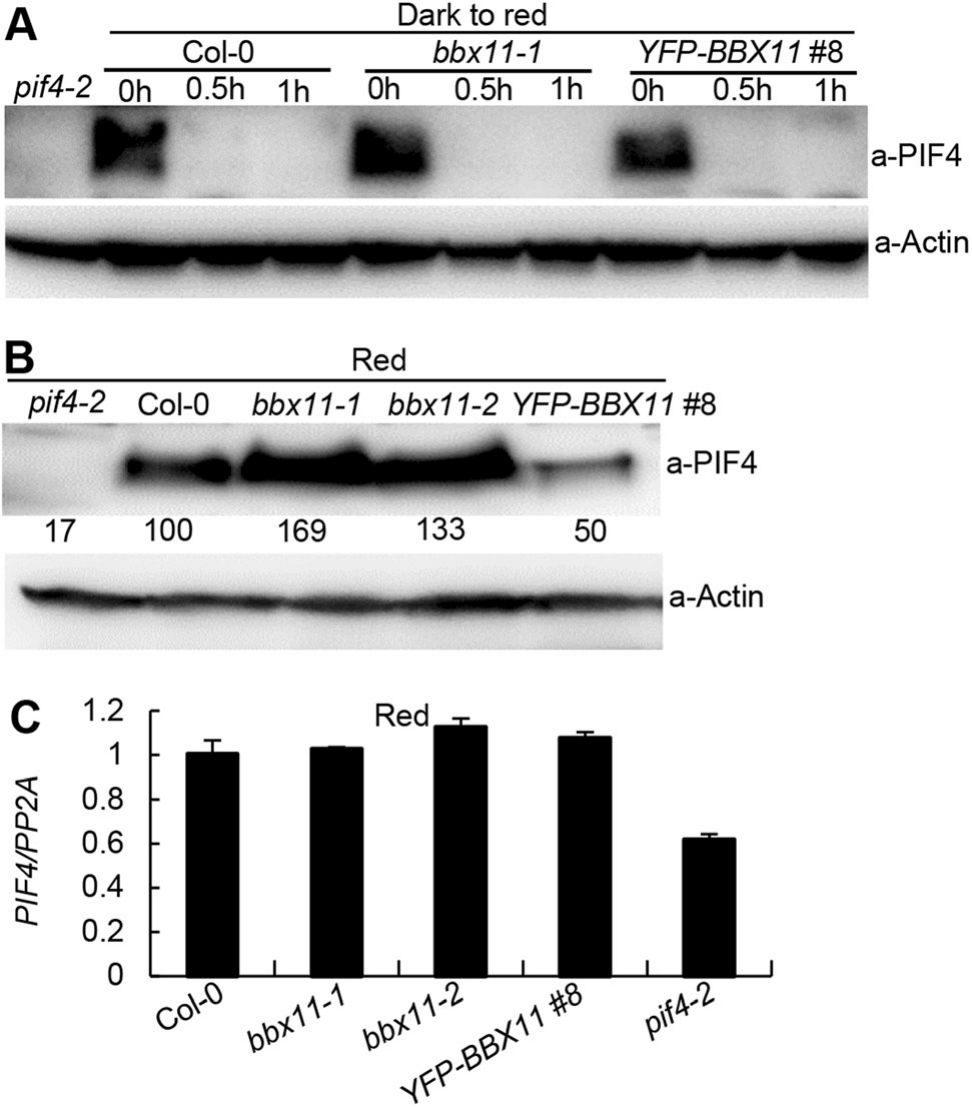
BBX11 represses the reaccumulation of PIF4 in the prolonged red light. **A** Immunoblots showing the PIF4 protein levels in Col-0, *bbx11-1* and *YFP-BBX11 #8* seedlings grown in darkness for 4 days, then transferred to red (94 μmol/m^2^/s) light as indicated time points (0, 0.5, and 1 h). *pif4-2* served as a negative control. Anti-Actin was used as a loading control. **B** Immunoblots showing the PIF4 protein levels in Col-0, *bbx11-1*, *bbx11-2*, *YFP-BBX11 #8* seedlings grown in the continuous red (94 μmol/m^2^/s) light for 4 days. *pif4-2* served as a negative control. Anti-Actin was used as a loading control. Numbers below the immunoblots indicate the relative intensities of PIF4 bands normalized to those of Actin, and the ratio was set to 100 for that in Col-0. **C** The transcription levels of *PIF4* in Col-0, *bbx11-1*, *bbx11-2*, *YFP-BBX11 #8* seedlings grown in the continuous red (94 μmol/m^2^/s) light for 4 days, as determined by real-time qRT-PCR. *pif4-2* served as a negative control

Red light activated-phyB promotes the rapid degradation of PIF4 (Huq and Quail 2002; Lorrain et al. 2008; Zhang et al. 2017a), we, therefore, examined the PIF4 protein levels in dark-grown *bbx11* single mutants and *BBX11* overexpressors upon transferred to red light for various time points (0, 0.5 and 1 h). Etiolated Col-0, *bbx11-1* and *YFP-BBX11 #8* seedlings accumulated comparable PIF4 proteins. Upon transferred to red light for 0.5 or 1 h, PIF4 proteins were barely detectable in all these seedlings (Fig. 3A), suggesting that BBX11 likely has a litter effect on the phyB- and red light-triggered rapid degradation of PIF4 (Fig. 3A). Following prolonged red light irradiation, PIF4 re-accumulates and maintains at a high level to promote seedling growth (Zhang et al. 2017a; Yan et al. 2020). We next investigated the PIF4 protein levels in Col-0, *bbx11-1*, *bbx11-2* and *YFP-BBX11 #8* seedlings grown in constant red light for 4 d. Two independent *bbx11* (*bbx11-1* and *bbx11-2*) mutant seedlings accumulated obviously more PIF4 abundance, whereas the PIF4 protein levels in *YFP-BBX11 #8* were clearly less abundant compared to those in Col-0, *bbx11-1* and *bbx11-2* (Fig. 3B), suggesting that BBX11 promotes the degradation of PIF4 in the prolonged red light.

As BBX11 acts as a transcription factor (Zhao et al. 2020), we thus examined whether BBX11 regulates the *PIF4* at the transcriptional level. The transcript levels of *PIF4* were not significantly altered in *bbx11-1*, *bbx11-2* and *YFP-BBX11 #8* compared to that in Col-0 grown in the constant red light, while the expression of *PIF4* was drastically decreased in the *pif4-2* mutant seedlings (Fig. 3C), suggesting that BBX11 may not affect the expression of *PIF4* in red light-grown seedlings.

### BBX11 enhances the interaction between phyB and PIF4

**Fig. 4:**
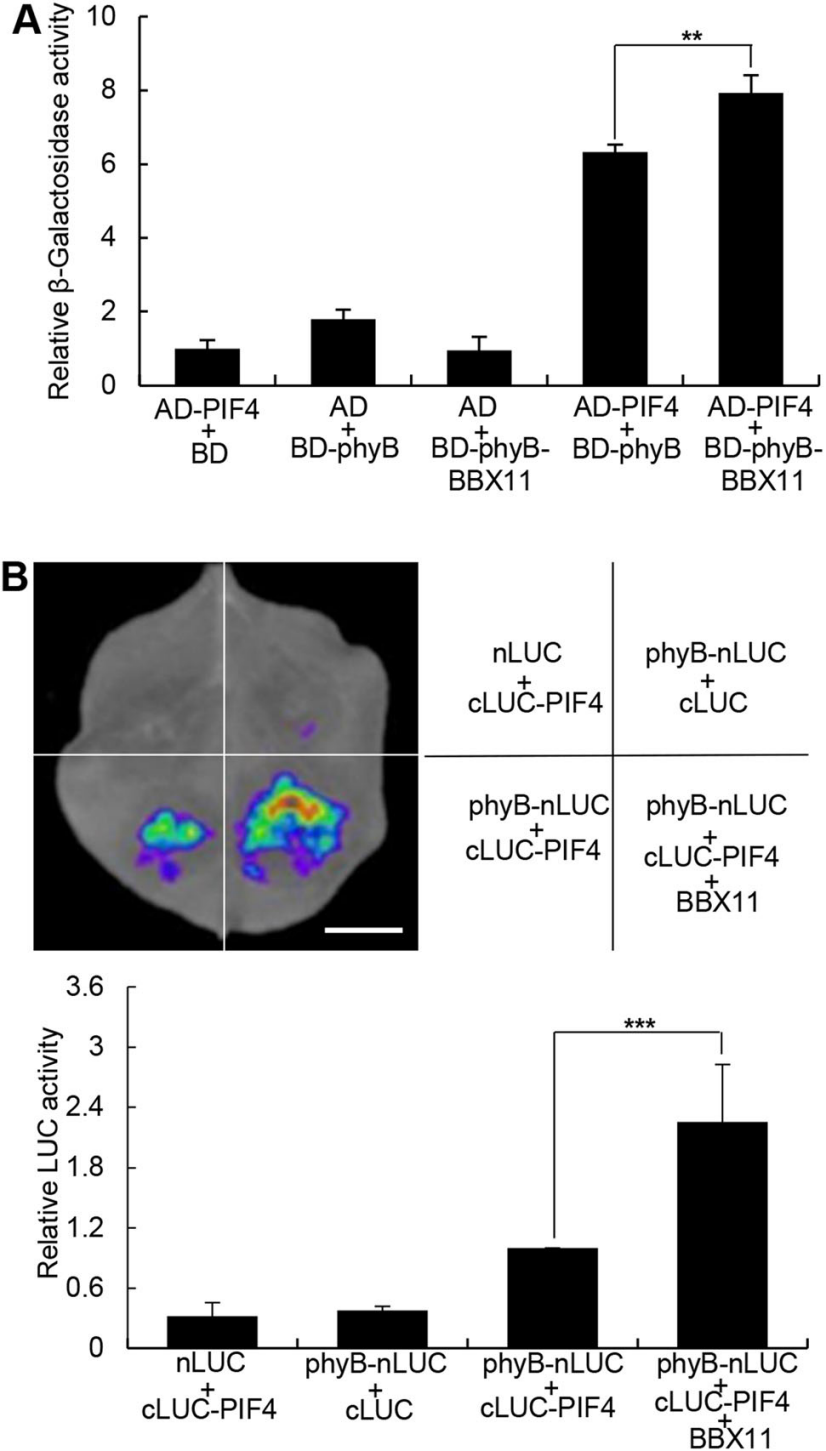
BBX11 enhances the interaction of phyB with PIF4. **A** Yeast-three hybrid assays showing that BBX11 enhances the interaction of phyB with BBX11 in yeast cells. **B** LCI assays showing that BBX11 enhances the interaction between phyB and PIF4 in living plant cells. Bar = 1 cm. Data are the means SD of three independent biological replecates. Asterisks represent statistically significant differences (**P < 0.01;*** P < 0.001), as determined by Student’s t-test

The interaction of phyB with PIF4 is required prior to the degradation of PIF4 upon red light irradiation (Lorrain et al. 2008). Considering that BBX11 not only interacts with both phyB and PIF4 but also promotes the degradation of PIF4 (Figs. 1, 2 and 3A), we carried out yeast-three hybrid assays to examine whether BBX11 affects the interaction of phyB with PIF4. phyB interacted with the PIF4 in yeast cells as revealed by relative β-Galactosidase activity (Fig. 4A), which is consistent with a previous study (Huq and Quail 2002). The value of relative β-Galactosidase activity was significantly increased in the presence of BBX11 (Fig. 4A). Next, we employed LCI assays to verify these results. The LUC signals were clearly detectable when transiently co-expressed phyB-nLUC and cLUC-PIF4 in the *Nicotiana benthamiana* leaves. While co-expression of phyB-nLUC and cLUC-PIF4 together with BBX11 in the same leaves led to a markedly increase in the LUC signals as detected in the same experimental system (Fig. 4B). Together, these results suggest that BBX11 enhances the interaction of phyB with PIF4 in both yeast and living plant cells.

### BBX11 represses PIF4 biochemical activity

**Fig. 5:**
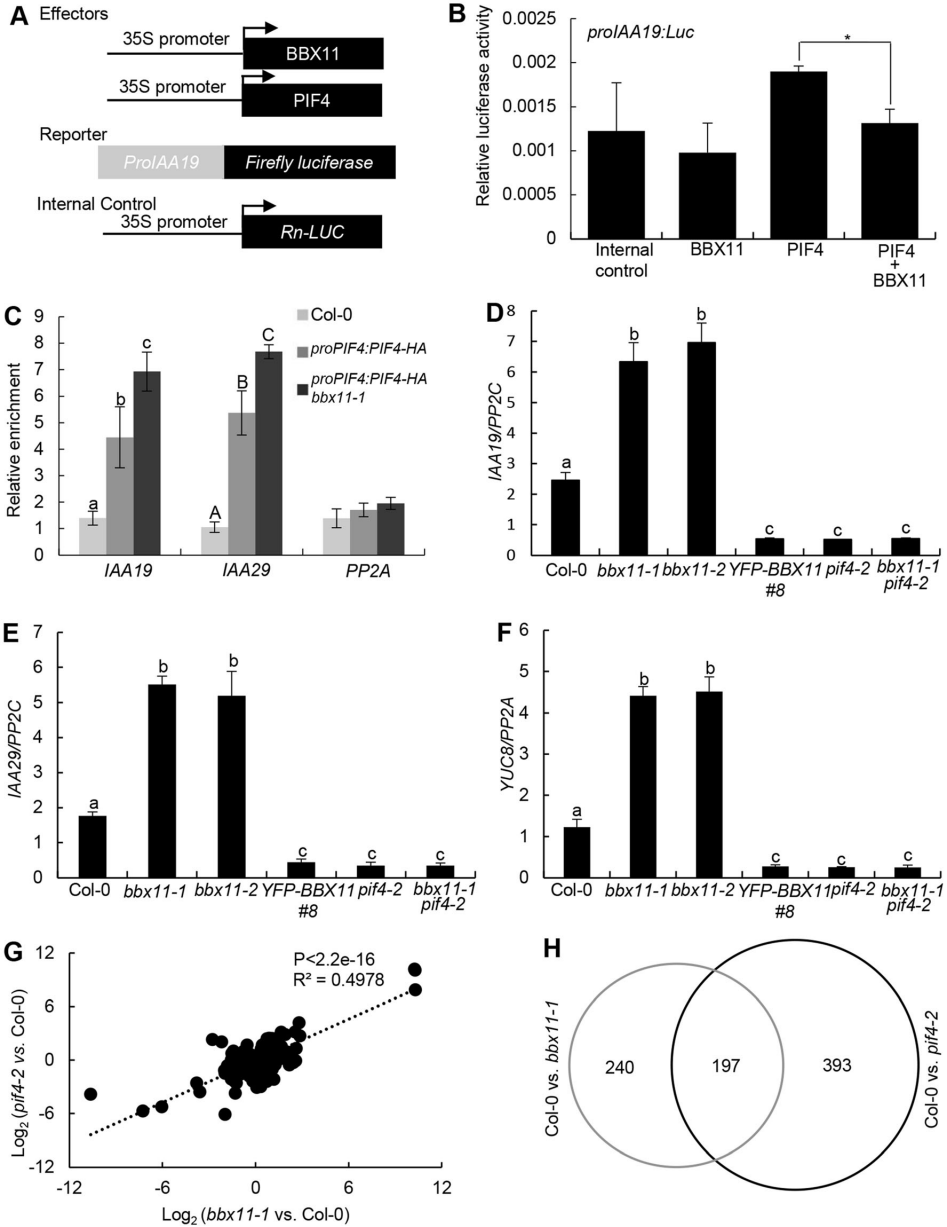
BBX11 represses the PIF4 binding to target sites. **A** Schematic representation of various constructs used in the transient transfection assay in *Nicotiana benthamiana* leaves. Arrow after the 35S promoter indicates the transcriptional start site. **B** Bar graphs showing that BBX11 represses the activation of the *proIAA19:LUC* by PIF4. Error bars represent SD of three independent transient transfections in Arabidopsis protoplasts. Asterisks represent statistically significant differences (*P < 0.05), as determined by Student’s *t* test. **C** ChIP-qPCR assays showing that BBX11 is required for the PIF4 binding to the *IAA19* and *IAA29* promoter regions in vivo. ChIP-qPCR assays were performed using 4-d-old Col-0, *proPIF4:PIF4-HA*, and *proPIF4:PIF4-HA bbx11-1*seedlings with anti-HA antibodies. Plants were grown in red (94 μmol/m^2^/s) light for 4 days. The data represent means SD of three biological repeats. as determined by one-way ANOVA with Tukey’s post hoc analysis. RT-qPCR analysis of *IAA19* (**D**), *IAA29* (**E**) and *YUC8* (**F**) transcript levels in Col-0, *bbx11-1*, *bbx11-2*, *YFP-BBX11 #8*, *pif4-2* and *bbx11-1 pif4-2* seedlings grown in red (94 μmol/m^2^/s) for 4 days. Three biological replicates, each with three technical repeats, were performed. The data represent means SD of three biological repeats. Letters above the bars indicate significant differences (*P* < 0.05), as determined by one-way ANOVA with Tukey’s post hoc analysis. **G** Scatterplot analysis of the fold change for genes showing differential expression either in *bbx11-1* or in *pif4-2* compared to Col-0. **H** Venn diagram showing the number and overlap of genes whose expression was changed in *bbx11-1* and *pif4-2* seedlings

Given that BBX11 physically interacts with PIF4, we wonder whether BBX11 has any effect on the PIF4 activity. The *Nicotiana benthamiana* leaves when transiently expressed PIF4 alone or together with BBX11 accumulated comparable PIF4 protein levels (Supplemental Fig. 4A). Consistent with previous findings (Yu et al. 2019), PIF4 was able to activate the *proIAA19:LUC* reporter in a transient transcriptional activation assay. Although BBX11 could not activate *proIAA19:LUC*, the activation on this reporter was significantly decreased when co-expressed PIF4 and BBX11 (Fig. 5A, B), suggesting that BBX11 inhibits the PIF4 transcriptional activation activity. We next performed chromatin immunoprecipitaiton (ChIP) experiments to assess whether BBX11 affects the PIF4 binding to target sites in plants. PIF4 associated with the *IAA19* and *IAA29* promoter regions, which was consistent with a previous study (Sun et al. 2013). The relative enrichment of PIF4 on *IAA19* and *IAA29* promoter regions was markedly increased in the *proPIF4:PIF4-HA bbx11-1* transgenic seedling compared to that in the *proPIF4:PIF4-HA* seedlings (Fig. 5C). Together, these findings suggest that BBX11 negatively affects the binding of PIF4 to its target DNA cis-element. We next examined the PIF4-HA protein levels in *proPIF4:PIF4-HA* and *proPIF4:PIF4-HA bbx11-1* seedlings grown in constant red light. *proPIF4:PIF4-HA bbx11-1* seedlings accumulated more PIF4-HA protein compared with *proPIF4:PIF4-HA* (Supplemental Fig. 4B), further supporting the notion that BBX11 promotes the degradation of PIF4 in the prolonged red light (Fig. 3B). These results suggest that the accumulated PIF4 in red light-grown *proPIF4:PIF4-HA bbx11-1* seedlings may also contribute to the increased binding of *IAA19* and *IAA29* promoter regions.

### BBX11 negatively regulates PIF4-controlled gene expression

Considering that BBX11 negatively affected PIF4 abundance as well as its DNA binding affinity (Figs. 3B; 5A–C), we thus examined the transcript levels of three PIF4-controlled genes in *bbx11* mutant and transgenic plants overexpressing *BBX11* grown in the constant red light. The expression of *IAA19*, *IAA29*, and *YUC8* were significantly up-regulated in *bbx11-1* and *bbx11-2*, but clearly down-regulated in *YFP-BBX11 #8, pif4-2* and *bbx11-1 pif4-2* (Fig. 5D–F), implying that BBX11 negatively mediates the expression of PIF4 target genes in a PIF4-dependent manner in *Arabidopsis* seedling grown in the red light.

To identify the potential target genes regulated by BBX11 or co-regulated by BBX11 and PIF4 at the whole genome level, we performed RNA sequencing (RNA-seq) using 4-d old red light-grown Col-0, *bbx11-1* and *pif4-2* seedlings. The expression of approximately 437 and 590 genes were significantly changed in *bbx11-1* and *pif4-2* compared to Col-0 respectively (Supplemental Table 1 and 2). Further analysis revealed that the transcription of 197 genes was significantly altered in both *bbx11-1* and *pif4-2* seedlings (Fig. 5G, H; Supplemental Table 3), indicating that BBX11 co-regulates a number of common target genes with PIF4 in red light-grown seedlings.

### *BBX11* genetically acts upstream of *PIF4*

**Fig. 6:**
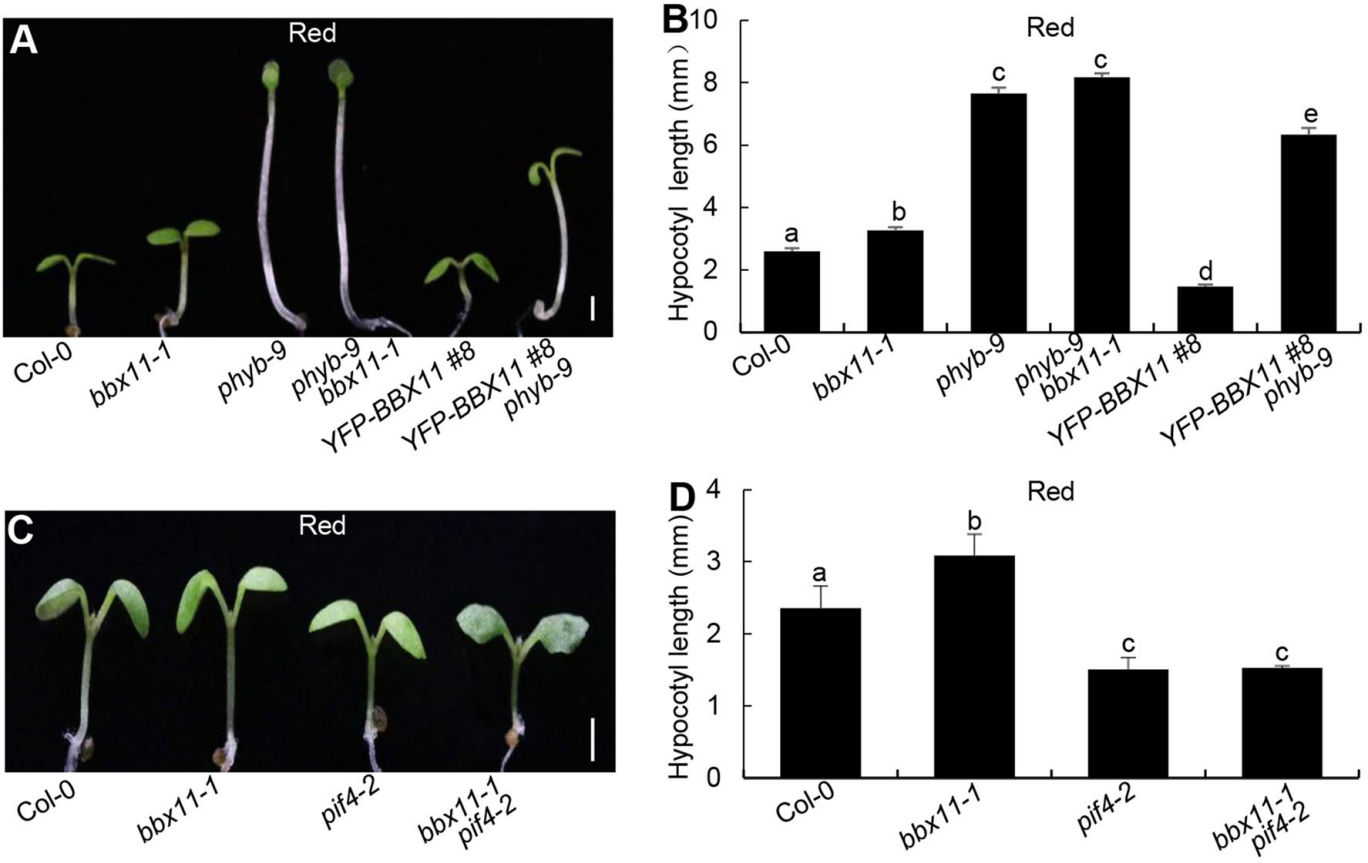
*BBX11* genetically acts upstream of *PIF4*. **A**, **B** Hypocotyl phenotype and length in 4-d-old Col-0, *bbx1-1*, *phyb-9*, *YFP-BBX11 #8* and *YFP-BBX11 #8 phyb-9* seedlings grown in red (94 μmol/m^2^/s) light conditions. **C**, **D** Hypocotyl phenotype and length in 4-day-old Col-0, *bbx1-1*, *pif4-2* and *bbx1-1 pif4-2* seedlings grown in the red (94 μmol/m^2^/s) light conditions. Hypocotyl length is expressed in millimeters. The data represent mean SE (*n* ≥ 60) of three biological replicates. Scale bars = 1 mm. Letters above the bars indicate significant differences (*P* < 0.05), as determined by one-way ANOVA with Tukey’s post hoc analysis

To examine the genetic links between *phyB*, *BBX11* and *PIF4*, we analyzed the hypocotyl phenotypes of various double mutants or transgenic seedlings grown in the red light. Consistent with results from previous studies (Neff and Chory 1998; Zhao et al. 2020), *bbx11-1* and *phyb-9* were longer than Col-0, whereas *YFP-BBX11 #8* was significantly shorter than Col-0 in the red light (Fig. 6A, B). The hypocotyl phenotypes of *phyb-9 bbx11-1* resembled those of *phyb-9* single mutant seedlings. *YFP-BBX11 #8 phyb-9* showed intermediate hypocotyls, which was longer than Col-0, *bbx11-1*, *YFP-BBX11 #8,* but shorter than *phyb-9* (Fig. 6A, B)*.* These genetic data imply that BBX11 functions in phyB signaling*. pif4-2* displayed shortened hypocotyl phenotypes (Fig. 6C, D), which was consistent with a previous study (Huq and Quail. 2002). The hypocotyl length of *bbx11-1 pif4-2* was indistigshible from that of *pif4-2* (Fig. 6C, D)*,* suggesting that BBX11 genetically acts upstream of PIF4 with respect to the hypocotyl growth in the red light.

## Discussion

phyB perceives the red light signals and regulates red light-dependent cellular and developmental processes in *Arabidopsis*. Upon red light exposure, inactive phyB pr form is converted into active pfr form that associates with PIFs including PIF4, subsequently triggering their rapid phosphorylation, ubiquitination and degradation (Huq and Quail 2002; Lorrain et al. 2008; Leivar and Monte 2014). As a b-HLH type transcription factor, PIF4 regulates a large number of genes’ expression to promote cell elongation and hypocotyl growth (Franklin et al. 2011; Sun et al. 2012, 2013; Jin et al. 2020). Thus phyB-PIF4 module acts as a signaling hub by which plants sense and transduce red light signals to control seedling development. Here, we have identified BBX11 as an essential regulator of this signaling hub. BBX11 negatively regulates PIF4 abundance and its activity through directly interacting with phyB and PIF4 in prolonged red light conditions.

Numerous studies have documented that a variety of BBXs plays critical roles in the control of photomorphogenesis in plants (Xu 2020; Song et al. 2020a). Of these, BBX4 is a positive regulator of red light signaling. phyB associates with BBX4 and stabilizes its abundance in the red light, thus allowing accumulated BBX4 to form heterodimers with PIF3 to inhibit its biochemical activity towards downstream target genes (Heng et al. 2019a). Same as BBX4, BBX11 promotes red light-mediated photomorphogenesis (Zhao et al. 2020; Job and Datta 2020). Upon red light illumination, BBX11 interacted with both phyB and PIF4 (Figs. 1 and 2). This modulation appears to result in the repression of PIF4 action through at least two distinct molecular regulatory mechanisms. (i) BBX11 acts as an adaptor in the interaction between phyB and PIF4, and facilitates the association of phyB with PIF4. Hence BBX11 may promote the phyB-triggered PIF4 degradation following red light irradiation (Figs. 3 and 4). (ii) BBX11 forms heterodimers with PIF4 and impairs its DNA binding ability or competes with PIF4 for DNA binding (Fig. 5A–C). These findings demonstrate that BBX11 not only promotes the degradation of PIF4 but also inhibits its biochemical activity, thereby limiting PIF4 action in regulating target genes’ expression and subsequent inhibition of hypocotyl growth. The hypocotyl length of *phyb-9 bbx11-1* was similar to that of *phyb-9*, and *YFP-BBX11 #8 phyb-9* displayed intermediate hypocotyl phenotypes in the red light. Moreover, the phenotypes of red light-grown *bbx11-1 pif4-2* resembled those of *pif4-2* single mutant seedlings (Fig. 6). These genetic data also support the conclusion that BBX11 acts in the phyB-PIF4-mediated signaling. Both BBX4 and BBX11 function as positive regulators of phyB signaling, however, they work in concert with distinct PIFs and exert different modes of action in promoting this process. The exact genetic and molecular interconnection between BBX4 and BBX11 awaits additional investigation.

It is well established that phyB inactive pr conformer is converted into pfr form, which translocates from the cytosol to the nucleus and interacts with PIF4 to induce its degradation (Huq and Quail 2002; Lorrain et al. 2008; Leivar and Monte 2014). These events may primarily occur at the early stage during the transition from dark to light, as phyB-imposed rapid PIF4 degradation implements within 10–60 min red light exposure (Lorrain et al. 2008; Zhang et al. 2017a; Yan et al. 2020). PIF4 re-accumulates to high abundant after prolonged (> 24 h) red light irradiation (Zhang et al. 2017a; Yan et al. 2020), suggesting that PIF4 is required and indispensable for promoting hypocotyl growth under prolonged red light conditions. The transcription factor MYB30, which is a negative regulator of photomorphogenesis, promotes the PIF4 re-accumulation under prolonged red light irradiation (Yan et al. 2020). By contrast, BBX11 promotes PIF4 degradation under the same conditions (Fig. 4). These facts suggest that MYB30 and BBX11 have opposite effects on the PIF4 re-accumulation following prolonged red light exposure. It is therefore of interest in future studies to explore the exact links between MYB30 and BBX11 in controlling PIF4 protein level.

BBX11 exclusively interacted with PIF4, but not with PIF1, PIF3 and PIF5 (Fig. 2), implying it has a specialized role on PIF4 in phyB signaling. PIF4 plays pleiotropic roles in various cellular, physiological and developmental processes such as miRNA biogenesis, stomatal development, photomorphogenesis, thermomorphogenesis, phototropic response, brassinosteroid signaling, auxin biogenesis and signaling (Franklin et al. 2011; Oh et al. 2012; Lau et al. 2018; Sun et al. 2012, 2013, 2018). Thus BBX11 may be also involved in these diverse developmental and signaling pathways via modulating PIF4 action. In addition to phys, CRYPTOCHROME 1 (CRY1), COP1, DE-ETIOLATED 1 (DET1), CIRCADIAN CLOCK-ASSOCIATED 1 (CCA1), ENHANCED PHOTOMORPHOGENIC 2(EPP2), LATE ELONGATED HYPOCOTYL (LHY), TIMING OF CAB 1(TOC1), ELF3-ELF4-LUX evening complex, COP1 SUPPRESSOR 4 (CSU4), HY5, COLD- REGULATED GENE 27 (COR27) and PIF4 itself regulate the PIF4 abundance, its transcriptional activity or transcription under specific conditions or at specific time points (Más et al. 2003; Nusinow et al. 2011; Nieto et al. 2015; Ma et al. 2016; Zhu et al. 2016, 2020; Huai et al. 2018; Zhao et al. 2018; Dong et al. 2020; Li et al. 2020; Zhai et al. 2020). Our study reveals that BBX11 is a novel negative regulator of PIF4. Most importantly, BBX11 plays dual role in inhibiting PIF4 function through promoting phyB-imposed PIF4 degradation as well as repressing the binding of PIF4 to its target sites. Altogether, these facts suggest a complex scenario whereby multiple factors converge on PIF4 in maintaining its appropriate abundance and/or transcriptional activation activity that ensures normal plant growth and development under natural conditions.

**Fig. 7:**
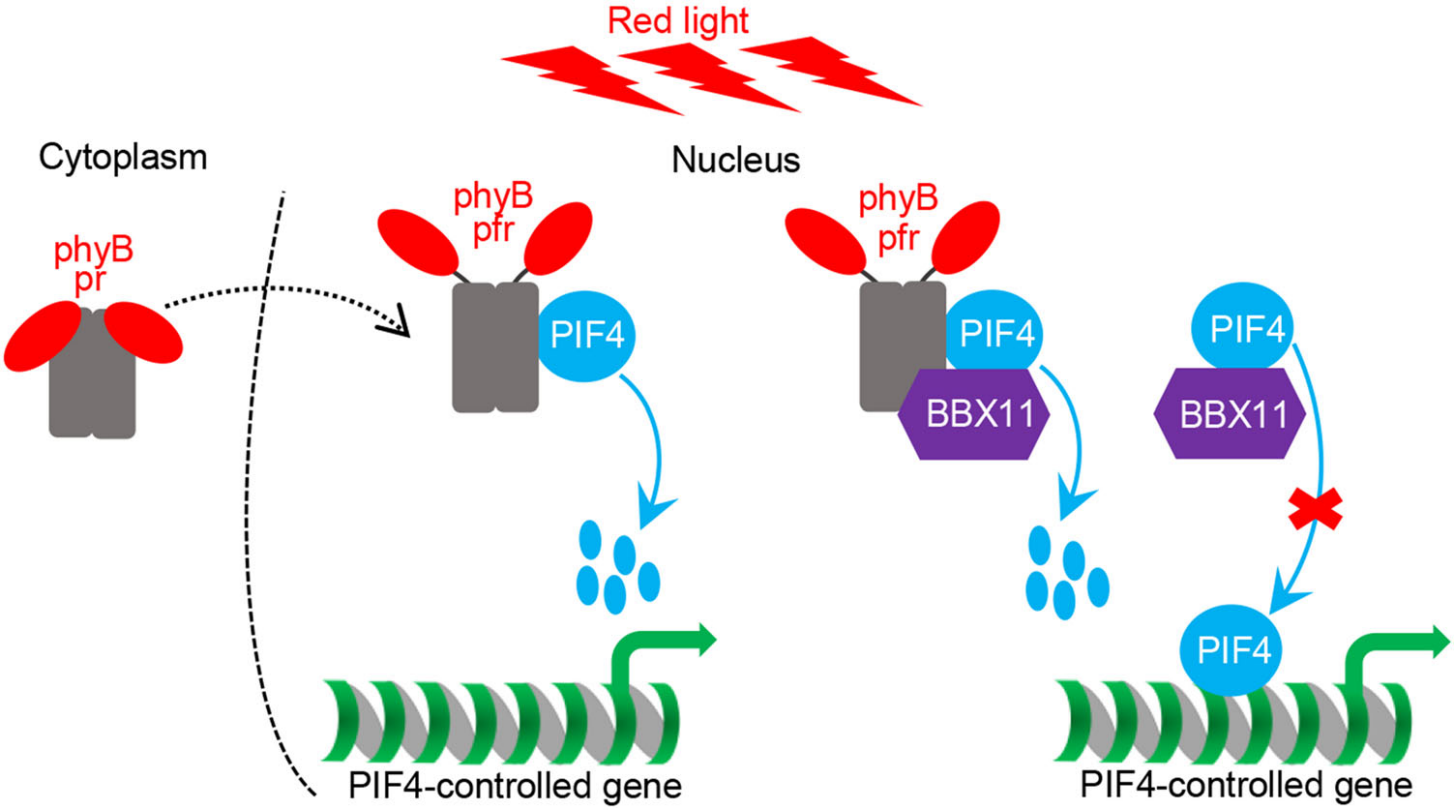
A working model depicting that how BBX11 represses PIF4 function in the red light. Upon red light irradiation, pr form of phyB converts into pfr form that interacts with PIF4 to trigger its rapid degradation via the 26S proteasome system. On one hand, BBX11 enhances the interaction of phyB with PIF4 to promote the degradation of PIF4. On the other hand, BBX11 inhibits the binding of PIF4 to target DNA cis-elements. Hence, BBX11 represses the PIF4 function and PIF4-controlled gene expression to promote photomorphogenesis

In summary, our results reveal that BBX11 is an essential regulator of the phyB-PIF4 signaling hub. BBX11 associates with both phyB and PIF4 that serves to enhance the interaction between phyB and PIF4, which in turn promoting the degradation of PIF4. On the other hand, BBX11 inhibits the DNA binding ability of PIF4. Consequently, these molecular events result in the repression of PIF4 function towards target genes (Fig. 7). Thus BBX11, acting downstream of phyB, negatively controls the PIF4 abundance as well as its biochemical activity in promoting photomorphogenesis in the red light. These observations demonstrate that the phyB-BBX11-PIF4 regulatory module acts as a central signaling hub that orchestrates red light-mediated seedling development.

## Materials and Methods

### Plant materials and growth conditions

The *phyb-9* (Neff and Chory 1998), *pif4-2* (Leivar et al. 2008), *bbx11-1*, *bbx11-2* mutants (Zhao et al. 2020), *YFP-BBX11 #8* (Zhao et al. 2020)*,* and *proPIF4:PIF4-HA* (Zhang et al. 2017a) transgenic lines are of Col-0 ecotype. Double mutants/transgenic plants were generated by genetic crossing, and homozygous lines were verified by PCR genotyping or antibiotic screen. Seeds were surface sterilized with 30% commercial Clorox bleach and sown on 1 × Murashige and Skoog (MS) medium containing 1% sucrose and 0.8% agar. The seeds were stratified in darkness for 3 days at 4 °C, then transferred to light chambers maintained at 22 °C. The fluence rates of the light growth chambers were 100 μmol/m^2^/s for white light and 94 μmol/m^2^/s for a red light.

### Plasmid construction

To generate *pB42AD-BBX11*, the full-length of *BBX11* were amplified by PCR with the respective pairs of primers and then cloned into the *EcoRI/XhoI* sites of the *pB42AD* vector (BD Clontech). To generate *pLexA-phyB, pLexA-phyB-N*, *pLexA-phyB-C*, *pLexA-phyB-C1, pLexA-phyB-C2, pLexA-BBX11, pLexA-BBX11(1–218), pLexA-BBX11 (1–97), pLexA-BBX11 (84–286), pLexA-BBX11 (280–332) and pLexA-BBX11Δ(97–213),* full-length *phyB, phyB-N*(*1–652*), *phyB-C(652–1172)*, *phyB-C1(910–1172)*, *phyB-C2(652–910),* full-length *BBX11, BBX11(1–218), BBX11(1–97), BBX11(85–286), BBX11(280–332)* and *BBX11* lacking *(98–212)* fragments were amplified by PCR with a specific pair of primers and then cloned into the *EcoRI/XhoI* sites of the *pLexA* vector (BD Clontech). *pB42AD-PIF1, pB42AD-PIF3, pB42AD-PIF4, pB42AD-PIF5* (Dong et al. 2014)*, pGreenII0800-proIAA19:LUC* (Yu et al. 2019), *pEarley Gate-35S:myc-BBX11* (Zhao et al. 2020) constructs were described previously. The primers used for plasmids construction were listed in Supplemental Table 4.

### Measurement of hypocotyl length

To measure the hypocotyl length of seedlings, seeds were surface sterilized and sown on MS plates. The seeds were stratified at 4 °C in darkness for 3 days, and then placed in continuous white (100 μmol/m^2^/s) light for 8 h to induce uniform germination. Then the seeds were transferred to continuous red (94 μmol/m^2^/s) light conditions and incubated at 22 °C for 4 days. The hypocotyl length of seedlings was measured using ImageJ software.

### Co-Immunoprecipitation (Co-IP) assay

Co-IP assays were performed as described previously (Lin et al. 2018). *Agrobacterium strain GV3101* cells carrying the 35S:myc-BBX11, 35S:phyB-Flag or 35S:PIF4-Flag constructs were transiently infiltrated into *Nicotiana benthamiana* leaves. The plants were grown under long-day conditions (16 h Light/8 h Dark) for 3 d and lysed. Extracts were incubated with 4 μL of anti-myc antibodies (1:250 v/v) (Sigma-Aldrich) coupled with 25 μL of Protein-A Sepharose (GE Healthcare) for 3 h at 4 °C. The Sepharose was washed three times using protein extraction buffer. The precipitates were eluted into 100 mM glycine (pH 2.5) and 100 mM NaCl, immediately neutralized by 2 M Tris–HCl (pH 9.0), and 100 mM NaCl, and then concentrated using StrataClean Resin (Stratagene) prior to immunoblot analysis.

### Immunoblot analysis

For immunoblot analysis, *Arabidopsis* seedlings were homogenized in protein extraction buffer containing 100 mM NaH_2_PO_4_, 10 mM Tris–HCl (pH8.0), 200 mM NaCl, 8 M Urea, 1 mM PMSF, and 1 × complete protease inhibitor cocktail (Roche). Primary antibodies used in this study were anti-PIF4 (Abicode Cat.R2534-4), anti-HA (MBL, Cat.M180-3), anti-myc (Sigma-Aldrich, Cat.M4439), and anti-Actin (Sigma-Aldrich, Cat.A0480).

### Yeast two-hybrid assays

Yeast two-hybrid assays were performed using the Matchmaker LexA Two-Hybrid System as described in the Yeast Protocols Handbook (BD Clontech). The respective combinations of *pLexA* and *pB42AD* fusion plasmids were co-transformed into yeast strain *EGY48* containing *p8op-LacZ* plasmid. The empty *pLexA* and *pB42AD* vectors were co-transformed in parallel as negative controls. Transformants were selected and grown on SD/–His–Trp–Ura dropout plates at 30 °C. The transformants were grown on SD/–His–Trp–Ura dropout plates containing 80 mg L^−1^ X-gal for blue color development.

### Chromatin immunoprecipitation (ChIP)

The ChIP assays were performed as described by Xu et al. (2016). Chromatin isolation was performed using Col-0, *proPIF4:PIF4-HA* and *proPIF4:PIF4-HA bbx11-1* transgenic seedlings grown under constant red (94 μmol/m^2^/s) light for 4 d. The resuspended chromatin was sonicated at 4 °C to 250- to 500-bp fragments. The sheared chromatin was immunoprecipitated, washed, reverse cross-linked, and finally amplified. About 10% of sonicated but nonimmunoprecipitated chromatin was reverse cross-linked and used as an input DNA control. Both immunoprecipitated DNA and input DNA were analyzed by real-time qPCR (Applied Biosystems). Monoclonal anti-HA antibody (MBL, Cat.M180-3), was used for the assays. All primers used for this assay are listed in Supplemental Table 4.

### Firefly LCI assay

The firefly LCI transient expression assay was performed as described previously (Chen et al. 2008). *Agrobacterium strain GV3101* strains carrying the binary plasmids of *pCAMBIA1300-phyB-nLUC* and *pCAMBIA1300-cLUC-BBX11 or pCAMBIA1300-PIF4-nLUC* and *pCAMBIA1300-cLUC-BBX11* were co-infiltrated into *Nicotiana benthamiana* leaves. The corresponding empty vector *pCAMBIA1300-nLUC* or *pCAMBIA1300-cLUC* was infiltrated as a negative control. After a 2 d incubation in darkness at 22 °C and an additional 1 d incubation under a 16-h light/8-h dark photoperiod, the leaves were harvested, and 1 mM D-luciferin (BD Monolight; BD Biosciences) solution was sprayed onto the tobacco leaves. Luciferase activity was measured with the LB 985 NightSHADE Spectrum imaging system (Berthold).

### Transient luciferase expression assays

*Nicotiana benthamiana* plants grown in LD conditions (16 h light/8 h dark) were used for transient transactivation assay. *Agrobacterium strain GV3101* cells carrying the 35S:myc-BBX11, 35S:PIF4-Flag or *proIAA19:LUC* constructs were transiently infiltrated into *Nicotiana benthamiana* leaves as indicated combinations. Firefly luciferase (LUC) and Renillia luciferase (Ren) were assayed using the Dual-Luciferase Reporter Assay System (Promega). The Ren gene driven by the cauliflower mosaic virus 35S promoter was used as the control. The relative activity was expressed as a ratio of LUC/Ren.

### RNA-sequencing (RNA-seq) analysis

Total RNA was extracted from the 4-d-old Col-0, *bbx11-1* and *pif4-2* seedlings grown in constant red light (94 μmol/m^2^/s) conditions. Then, mRNA sequencing libraries were constructed, and sequencing was performed using the Illumina HiSeq 2500 platform according to the manufacturer’s instruction (HiSequation 2500 user guide) by Shanghai Hanyu-Bio. Three independent biological replicates were performed. RNA-Seq reads were mapped to *Arabidopsis* TAIR 10 using Hisat2 (version 2.05; Kim et al. 2015) software using default parameters. Then, raw reads for each gene were calculated by HTseq (Anders et al. 2015) before calculating differential gene expression. Then, differentially expressed genes among two conditions were identified using the general liner models method in the edgeR package (version 3.12.0; Robinson et al. 2010) with a false positive rate of 0.05 and a fold-change of 2.

### Total RNA isolation and quantitative qRT-PCR

4-d-old *Arabidopsis* seedlings grown under red light (94 μmol/m^2^/s) conditions were used to isolate total RNA with an RNeasy Plant Mini Kit (Qiagen). cDNA synthesis reactions were performed with a 5 × All-In-One RT MasterMix (Applied Biological Materials) according to the manufacturer’s instructions. cDNA templates and primer sets were mixed with Hieff qPCR SYBR Green Master Mix (Yeasen), and real-time qPCR was performed on a StepOnePlus Real-time qPCR detection system (Applied Biosystems). Each experiment was performed at least three times with similar results and three technical replicates were performed for each sample. The expression levels were normalized to that of housekeeping gene *PP2A*. The primers used in RT-qPCR are listed in Supplemental Table 4.

### Statistical analysis

Statistical analysis was performed using Microsoft Excel, GraphPad Prism version 5.0 or through an online website (http://astatsa.com/OneWay_Anova_with_TukeyHSD/).

### Accession numbers

Sequence data from this article can be found in the *Arabidopsis* Genome Initiative database or the GenBank/EMBL libraries under the following accession numbers: *phyB* (*AT2G18790*); *PIF4* (*AT*4G25350); BBX11 (*AT2G47890*). RNA-seq data have been deposited into the Gene Expression Omnibus with the following accession number: GSE166109.

## Supplementary Information

Below is the link to the electronic supplementary material.

Figure 1. phyB and its PRD domain exhibit self-transactivation activity in yeast cells. Figure 2. The middle portion of BBX11 is responsible for its self-transactivation activity in yeast cells. Figure 3. phyB may not affect the BBX11 abundance in the red light. Figure 4. PIF4 protein levels in the proPIF4:PIF4-HA and proPIF4:PIF4-HA bbx11-1 seedlings grown in the red light

Table 1. List of 437 BBX11-regulated genes

Table 2. List of 590 PIF4-regulated genes

Table 3. List of 197 BBX11 and PIF4-coregulated genes

Table 4. Primers used in this study
