# Supplementary material for: BBX11 promotes red light-mediated photomorphogenic development by modulating phyB-PIF4 signaling
Source: aBIOTECH. 2021 Apr 26;2(2):117–30. doi: 10.1007/s42994-021-00037-2 (PMC9590482; doi:10.1007/s42994-021-00037-2)
Supplement: Supplementary file 1 — Figure 1. phyB and its PRD domain exhibit self-transactivation activity in yeast cells. Figure 2. The middle portion of BBX11 is responsible for its self-transactivation activity in yeast cells. Figure 3. phyB may not affect the BBX11 abundance in the red light. Figure 4. PIF4 protein levels in the proPIF4:PIF4-HA and proPIF4:PIF4-HA bbx11-1 seedlings grown in the red light [file 42994_2021_37_MOESM1_ESM.pdf]

Supplemental Information

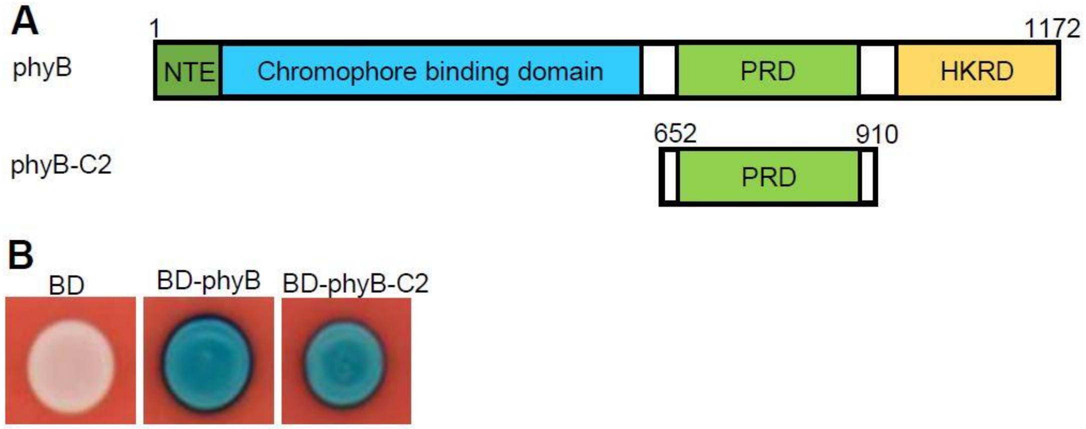

Supplemental Figure 1. phyB and its PRD domain exhibit self-transactivation activity in yeast cells

(A) Schematic diagram of phyB and phyB-C2 constructs used in the self-transactivation assays in yeast. Numbers indicate the amino acid positions in phyB.

(B) BD-phyB and BD-phyB-C2 show self-transactivation activity in yeast cells. The empty vector pLexA-BD served as a negative control.

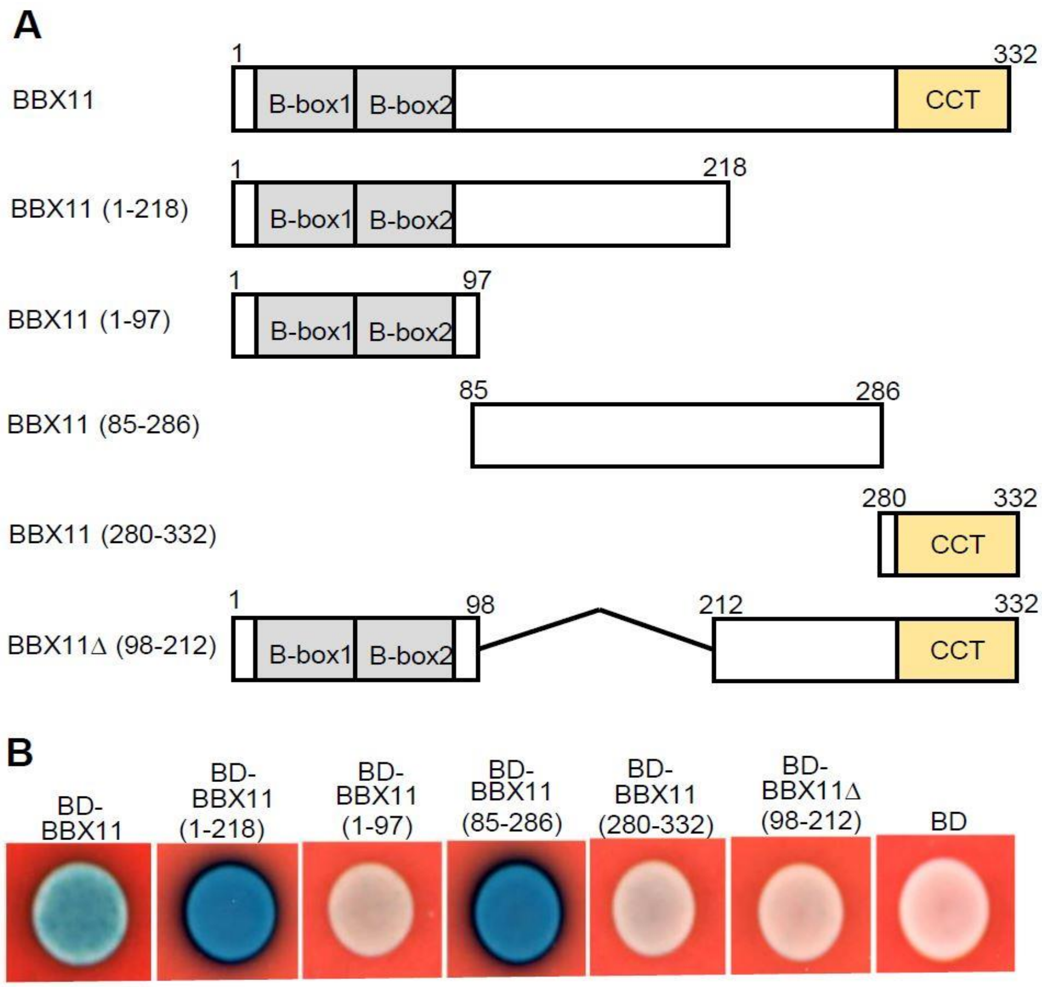

Supplemental Figure 2. The middle portion of BBX11 is responsible for its self-transactivation activity in yeast cells

(A) Schematic diagram of various constructs used in the self-transactivation assays in yeast. Numbers indicate the amino acid positions in BBX11.

(B) BD-BBX11, BD-BBX11 (1-218) and BD-BBX11 (85-286), but not BD-BBX11 (1-97), BD-BBX11 (280-332), and BD-BBX11Δ (98-212) show self-transactivation activity in yeast cells. The empty vector pLexA-BD served as a negative control.

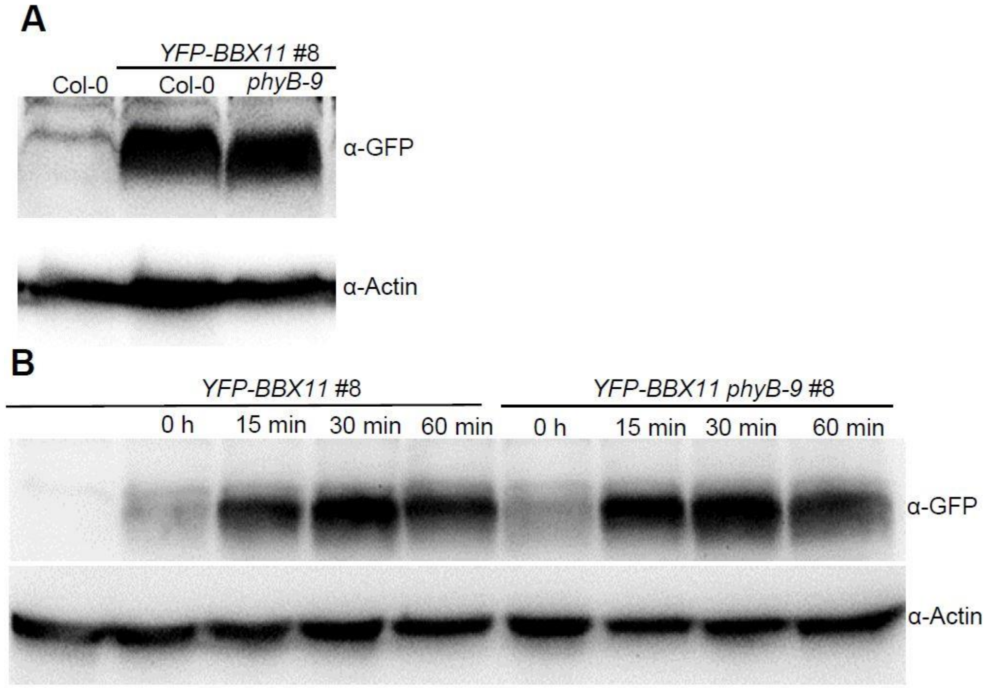

Supplemental Figure 3. phyB may not affect the BBX11 abundance in the red light

(A) Immunoblots showing the YFP-BBX11 protein levels in *YFP-BBX11 #8*, and *YFP-BBX11 #8 phyb-9* seedlings grown in the continuous red (94 μmol/m^2/s) light for 4 d.

(B) Immunoblots showing the YFP-BBX11 protein levels in 4-d-old dark-grown *YFP-BBX11 #8*, and *YFP-BBX11 #8 phyb-9* seedlings upon transferred to the red (94 μmol/m^2/s) light for the indicated time periods (0, 15, 30 and 60 min).

Col-0 served as a negative control. Anti-Actin was used as a loading control.

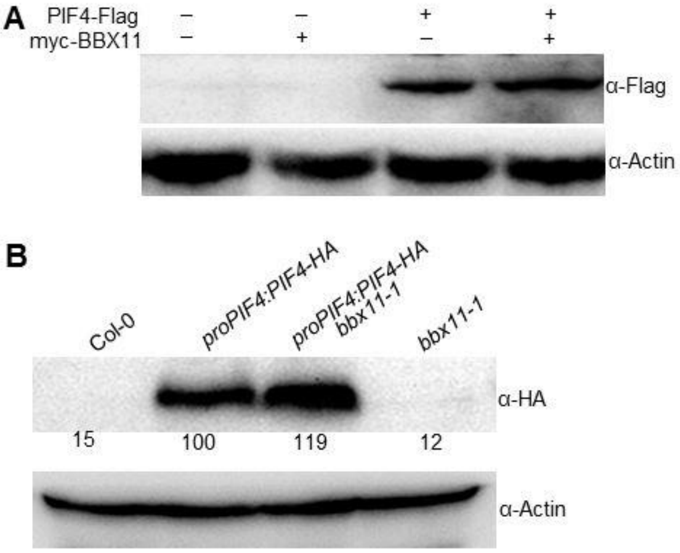

Supplemental Figure 4. PIF4 protein levels in the *proPIF4:PIF4-HA* and *proPIF4:PIF4-HA bbx11-1* seedlings grown in the red light

(A) Immunoblots showing the PIF4-Flag protein levels in *Nicotiana benthamiana* leaves when transiently expressed PIF4-Flag alone or together with myc-BBX11. Anti-Actin was used as a loading control. (Supports Figure 5A-B)

(B) Immunoblots showing the PIF4-HA protein levels in 4-d-old red light (94 μmol/m^2/s) grown *proPIF4:PIF4-HA* and *proPIF4:PIF4-HA bbx11-1* seedlings. Col-0 and *bbx11-1* served as a negative control. Anti-Actin was used as a loading control. Numbers below the immunoblots indicate the relative intensities of PIF4 bands normalized to those of Actin, and the ratio was set to 100 for that in Col-0. (Supports Figure 5C)
