# Supplementary material for: BBX11 promotes red light-mediated photomorphogenic development by modulating phyB-PIF4 signaling
Source: aBIOTECH. 2021 Apr 26;2(2):117–30. doi: 10.1007/s42994-021-00037-2 (PMC9590482; doi:10.1007/s42994-021-00037-2)
Supplement: Supplementary file 5 — Table 4. Primers used in this study [file 42994_2021_37_MOESM5_ESM.docx]

**Supplemental Table 4. Primers used in this study**

| **Primer's name** | **Primer sequences (5’→3’)**  **Underscored nucleotides indicate restriction sites for cloning** | **Construct's name** |
| --- | --- | --- |
| BBX11-F | CCCGAATTC ATGGAAGCAGAAGAAGGTCA | *pB42AD-BBX11* |
| BBX11-R | CTTCTCGAG TCATGGATCTGCTGCCTTGG |  |
| phyB-F | AACGGCGACTGGCTGGAATTCATGGTTTCCGGAGTCGGGGGTAGTG | *pLexA-phyB* |
| phyB-R | TTGGCTGCAGGTCGACTCGAGCTAATATGGCATCATCAGCATCATG |  |
| phyB-N-F | AACGGCGACTGGCTGGAATTCATGGTTTCCGGAGTCGGGGGTAGTG | *pLexA-phyB-N* |
| phyB-N-R | TTGGCTGCAGGTCGACTCGAGGCACCTAACTCATCAATCCCCTGT |  |
| phyB-C-F | AACGGCGACTGGCTGGAATTCATGAACTCTAAAGTTGTGGATGGT | *pLexA-phyB-C* |
| phyB-C-R | TTGGCTGCAGGTCGACTCGAGCTAATATGGCATCATCAGCATCATG |  |
| phyB-C1-F | AACGGCGACTGGCTGGAATTCATGAACTCTAAAGTTGTGGATGGT | *pLexA-phyB-C1* |
| phyB-C1-R | TTGGCTGCAGGTCGACTCGAGCTCAGGGCTCGGGATTTGCAAGAAA |  |
| phyB-C2-F | AACGGCGACTGGCTGGAATTCCTGCAGCAAGCTTTAGCAGTCCAA | *pLexA-phyB-C2* |
| phyB-C2-R | TTGGCTGCAGGTCGACTCGAGCTAATATGGCATCATCAGCATCATG |  |
| BBX11-F | CTGGAATTC ATGGAAGCAGAAGAAGGTCA | *pLexA-BBX11* |
| BBX11-R | CGACTCGAG TCATGGATCTGCTGCCTTGG |  |
| BBX11(1-218)-F | CTGGAATTC ATGGAAGCAGAAGAAGGTCA | *pLexA-BBX11(1-218)* |
| BBX11(1-218)-R | CGACTCGAG TCAGAACCATTTCAGTCCACTC |  |
| BBX11(1-97)-F | CTGGAATTC ATGGAAGCAGAAGAAGGTCA | *pLexA-BBX11(1-97)* |
| BBX11(1-97)-R | CGACTCGAG TCATTCAAAGGGTCTGCGGCTA |  |
| BBX11(85-286)-F | CTGGAATTC ATGACCGCCTCTTCCTCCCTTC | *pLexA-BBX11(85-286)* |
| BBX11(85-286)-R | CGACTCGAG TCACTCAAGACTGTTGATCTCATG |  |
| BBX11(280-332)-F | CTGGAATTC ATGCATGAGATCAACAGTCTTGAGAG | *pLexA-BBX11(280-332)* |
| BBX11(280-332)-R | CGACTCGAG TCATGGATCTGCTGCCTTGG |  |
| BBX11 (1-97)-F | CTGGAATTC ATGGAAGCAGAAGAAGGTCA | *pLexA-BBX11∆ (98-212)* |
| BBX11 (1-97)-R | TTCAAAGGGTCTGCGGCTA |  |
| BBX11 (213-332)-F | TAGCCGCAGACCCTTTGAAAGTGGACTGAAATGGTTC |  |
| BBX11 (213-332)-R | CGACTCGAG TCATGGATCTGCTGCCTTGG |  |
| phyB-nLUC-F | acgggggacgagctcggtaccATGGTTTCCGGAGTCGGGGGTA | *pCAMBIA1300-phyB-nLUC* |
| phyB-nLUC-R | cgcgtacgagatctggtcgacATATGGCATCATCAGCATCATGTC |  |
| BBX11-cLUC-F | tacgcgtcccggggcggtaccATGGAAGCAGAAGAAGGTCA | *pCAMBIA1300-cLUC-BBX11* |
| BBX11-cLUC-R | acgaaagctctgcaggtcgacTCATGGATCTGCTGCCTTGG |  |
| PIF4-nLUC-F | acgggggacgagctcggtaccATGGAACACCAAGGTTGGAGT | *pCAMBIA1300-PIF4-nLUC* |
| PIF4-nLUC-R | cgcgtacgagatctggtcgacAGTGGTCCAAACGAGAACCGTC |  |
| **Real-time qPCR** |  | |
| IAA19(F) | GCCAGGTGGTGGGGTGGCCACCG | |
| IAA19(R) | GGCCACACCGATGCCACGGAAAC | |
| IAA29(F) | CACGGCGATGAACAACAACATAT | |
| IAA29(R) | CTCTGTCGCAATCTTCATATTCG | |
| YUC8(F) | TGTATGCGGTTGGGTTTACGAGGA | |
| YUC8(R) | CCTTGAGCGTTTCGTGGGTTGTTT | |
| PIF4(F) | CCAGATCATCTCCGACCGGTTTG | |
| PIF4(R) | CTAGTGGTCCAAACGAGAACCGT | |
| PP2A(F) | TATCGGATGACGATTCTTCGTGCAG | |
| PP2A(R) | GCTTGGTCGACTATCGGAATGAGAG | |
| **ChIP-qPCR** | | |
| IAA19-ChIP(F) | GATATCAAATGACTCCACGTGTC | |
| IAA19-ChIP (R) | TCCGTGAAAGCTCTCTTCTTCAT | |
| IAA29-ChIP(F) | CTCACTACCTTTCGCTTAACGTG | |
| IAA29-ChIP(F) | GTAGTATGTAAATCTAGACCCAA | |
| PP2A(F) | TATCGGATGACGATTCTTCGTGCAG | |
| PP2A(R) | GCTTGGTCGACTATCGGAATGAGAG | |
